# Psychological Aspects Associated With Fertility Preservation in Oncology: An Exploratory Study

**DOI:** 10.3389/fpsyg.2020.608651

**Published:** 2020-12-22

**Authors:** Valentina Elisabetta Di Mattei, Gaia Perego, Paola Maria Vittoria Rancoita, Paola Taranto, Letizia Carnelli, Giorgia Mangili, Veronica Sarais, Alice Bergamini, Massimo Candiani

**Affiliations:** ^1^Division of Neuroscience, Clinical and Health Psychology Unit, IRCCS San Raffaele Scientific Institute, Milan, Italy; ^2^School of Psychology, Vita-Salute San Raffaele University, Milan, Italy; ^3^Department of Psychology, University of Milano-Bicocca, Milan, Italy; ^4^University Centre for Statistics in the Biomedical Sciences (CUSSB), Vita-Salute San Raffaele University, Milan, Italy; ^5^School of Medicine, Vita-Salute San Raffaele University, Milan, Italy; ^6^Obstetrics and Gynecology Unit, IRCCS San Raffaele Scientific Institute, Milan, Italy

**Keywords:** anxiety, defense mechanisms, depression, fertility preservation, oncology, personality, REM-71, TCI-R

## Abstract

**Objective:**

Gonadotoxicity is considered one of the most distressing side effects of cancer treatment. Although fertility preservation can be a valid solution, it also involves a challenging process. A clear understanding of the features of women who decide to pursue fertility preservation after cancer diagnosis is missing. The purpose of the present study was therefore to analyze the personality profile of female patients referred to oncofertility prior to gonadotoxic treatment.

**Methods:**

Fifty-two female cancer patients took part in the study. The Temperament and Character Inventory-Revised (TCI-R), the Response Evaluation Measure-71 (REM-71), the Beck Depression Inventory (BDI-II), and the State-Trait Anxiety Inventory-Y Form (STAI-Y) were administered to examine personality characteristics, defense mechanisms, depression and anxiety symptoms.

**Results:**

Compared with reference data of the Italian population, our sample reported significantly lower scores in Harm Avoidance and trait anxiety, and significantly higher levels of mature defense mechanisms. Most of the patients reported low scores in immature defense mechanisms, depression, and trait anxiety, and medium scores in state anxiety.

**Conclusions:**

Our findings suggest that these women display functional personality traits and defensive style, in association with low levels of depression and trait anxiety. These features may enable a proactive attitude to cancer and the ability to make long-term plans. This may favor psychological adjustment to cancer and a projection toward the future.

## Introduction

Remarkable advancements in cancer diagnosis and treatment have redefined oncologists’ focus from a treatment-based strategy to a wider view that includes survival and quality of life. Women consider potential loss of fertility as one of the most distressing late effects of cancer treatment (Crawshaw, 2013). Indeed, ovarian reserve may be impaired by surgical removal of reproductive organs, gonadotoxic chemotherapy treatments, or radiotherapy over reproductive organs. In order to compensate these negative consequences, cryopreservation of embryos, oocytes, or ovarian tissue is proposed to women to preserve their fertility.

The possibility of having children after cancer can be a powerful stimulus for recovery (Hershberger et al., 2013b), as it symbolizes the opposite of cancer, representing at first glance a promising option (Tschudin and Bitzer, 2009). However, fertility preservation can be a challenging process, as it can take up to 3 weeks in female patients, delaying oncological treatment (Logan et al., 2018). Indeed, an adequate organization of an Oncofertility Unit can reduce the time required by the procedure, encouraging consultants and patients to preserve fertility before gonadotoxic treatments (Sigismondi et al., 2015; Mangili et al., 2017) to shorten the time for oocyte cryopreservation and start anticancer treatment on time. In addition, women may be overwhelmed by all the difficult decisions and medical procedures they are required to undergo while fighting cancer. Thus, it is not surprising that fertility preservation rates remain quite low (Hershberger et al., 2013b). The literature points out several factors that influence this decision-making process, including personal factors (e.g., Peate et al., 2011; Hill et al., 2012; Kim et al., 2012; von Wolff et al., 2016), cancer-related clinical variables (e.g., Kim et al., 2012; von Wolff et al., 2016), childbearing attitudes (e.g., Hill et al., 2012; Hershberger et al., 2016) and cryopreservation-related factors (e.g., Kim et al., 2013; Baysal et al., 2015; Panagiotopoulou et al., 2018). However, the studies mentioned have produced mixed results, thus revealing inconsistent findings (Melo et al., 2019).

Therefore, a clear understanding of the features of the women who decide to pursue fertility preservation is missing, particularly focusing on patients’ decision rather than the actual feasibility of treatment (Melo et al., 2019). In particular, the choice of undergoing fertility preservation can be analyzed within the framework of the adaptation process to disease. In fact, this option can subtend a better adjustment to cancer, in so far as it implies a projection toward the future and a concern about one’s own quality of life. Stanton et al. (2007) identified the safeguard of life goals and the perception of personal growth as crucial indicators of adjustment to chronic conditions. In particular, the ability to manage such a complex situation may be influenced by personality traits, including Self-Directedness, Reward Dependence and Harm Avoidance (Bonacchi et al., 2012; Honorato et al., 2017).

In addition, defense mechanisms might play a role with respect to the adaptation to physical illness (Di Mattei et al., 2015). As cancer generates strong emotions, the mobilization of defenses is one of the main tools that is available to the patient to contain unpleasant feelings and to accept the current situation, excluding intolerable and painful experiences from awareness. The use of a wide range of flexible defenses contributes to protect the patient from fear and discomfort caused by the medical diagnosis, even increasing the chances of survival over time (Beresford et al., 2006). Moreover, defense style has been found to influence quality of life in oncological patients (Paika et al., 2010; Hyphantis et al., 2011, 2013).

In spite of the role played by personality features and defensive functioning in the adjustment to a disease, no studies have taken into account these characteristics in women who undergo fertility preservation techniques following cancer diagnosis. Thus, this study aimed to better understand the personality profile and defense style of female patients referred to an Oncofertility Unit after cancer diagnosis and the subsequent proposal of gonadotoxic treatment. In particular, we assessed temperament and character according to Cloninger’s biosocial theory of personality (Cloninger, 1999). In line with previous studies (Bonacchi et al., 2012; Honorato et al., 2017), we expected to find high levels of Self-Directedness (i.e., responsibility, hope, self-acceptance, self-actualization, and resourcefulness) and Reward Dependence (i.e., sensitivity, dedication, sociability, and ability to express affection and communicate), and low levels of Harm Avoidance (i.e., the ability to relax, courage, calm, optimism, even in situations that usually worry other people) (Cloninger, 1999). We hypothesized that these features could facilitate the planning of fertility preservation, despite the significant challenges associated with cancer. As mood and anxiety can interfere the assessment of temperament and character, particularly Harm Avoidance levels (Sato et al., 2001; Jiang et al., 2003), we controlled for these variables, assessing symptoms associated with depression and state and trait anxiety. In addition, we assessed defense mechanisms; in light of the studies showing that a mature defense style promotes a better adjustment to disease (Di Giuseppe et al., 2018), we expected to find a greater use of mature mechanisms in our sample of patients (i.e., defenses that attenuate distressing reality, without distorting it – Prunas et al., 2014).

## Materials and Methods

### Participants

Female cancer patients referred to the Oncofertility Unit of the San Raffaele Hospital in Milan after the proposal of gonadotoxic treatment between January 2014 and May 2016 were recruited to participate in the study. The Oncofertility Unit of the San Raffaele Hospital is an Italian reference center for fertility preservation in oncology; therefore, patients are referred here both within the hospital and from other hospitals in Italy. For this reason, they are usually already motivated to undergo fertility preservation. Additional eligibility criteria were the following: being at least 18 years old; speaking and understanding Italian; agreeing to voluntarily participate in the study through written informed consent. Patients were informed about the objectives of the study by a psychologist during the counseling session prior to the medical appointment, where a gynecologic oncologist and a reproductive gynecologist evaluated the patient in order to decide whether or not to refer her to pursue fertility preservation options (i.e., oocyte cryopreservation, ovarian tissue cryopreservation). Participants were asked to return questionnaires before the end of the fertility preservation process, which usually lasts 2 weeks.

Of the sixty-seven patients referred to the Oncofertility Unit, 15 women refused to participate or returned uncomplete questionnaires, giving a response rate of 77.61%. The final sample consisted of 52 patients.

The study was carried out following the guidelines of the Hospital Ethics Committee, which approved the protocol N. 149/INT/2019, in accordance with the Declaration of Helsinki.

### Measures

Patients were asked to complete a battery of self-administered tests which included:

(1) A self-report questionnaire purposely created for collecting socio-demographic (age, marital status, parity, educational level, occupation) and clinical (diagnosis, type of treatment–i.e., surgery-, previous miscarriages) characteristics.
(2) The Temperament and Character Inventory-Revised (TCI-R) (Cloninger, 1999) is based on Cloninger’s model of personality, which identifies four dimensions of temperament (Novelty Seeking: NS; Harm Avoidance: HA; Reward Dependence: RD; and Persistence: PS) and three dimensions of character (Self-Directedness: SD; Cooperativeness: CO; and Self-Transcendence: ST). High scores of HA denote the tendency of the person to behavioral avoidance in the face of potentially dangerous stimuli and to show negative effects; NS refers to exploratory behaviors and activation in response to novel stimuli; RD refers to social and affective abilities; P characterizes industrious, hard-working and stable individuals; SD expresses the competence of the individual toward autonomy, reliability and maturity; C relates to social skills, such as support, collaboration, partnership; ST denotes the aptitude toward mysticism, religion and idealism. It is composed of 240 items on a five-point Likert scale (1 = *definitely false* to 5 = *definitely true*). The Italian version of the questionnaire (Fossati et al., 2007), which was used in this study, demonstrated an adequate internal consistency, with Cronbach’s alpha values ranging from 0.79 to 0.91 for the main TCI-R dimensions. Test–retest reliability range from 0.76 to 0.88 (Martinotti et al., 2008). Normal scores for the Italian population were converted to *T* scores and grouped into five categories: significantly low (<30); low (30–39); medium (40–60); high (61–70); significantly high (>70). For each dimension, the corresponding cut-offs of the raw scores were also reported by Martinotti et al. (2008).
(3) The Response Evaluation Measure-71 (REM-71) (Steiner et al., 2001) assesses defense mechanisms in adults and adolescents. It is composed of 71 items, with each item scored on a nine-point Likert scale (from *strongly disagree* to *strongly agree*). Factorial analysis allowed for the identification of two factors based on the level of maturity of these defense mechanisms. Factor 1 (F1) expresses the global score regarding the immature defense mechanisms that can distort reality, contributing to less adaptive functioning. This factor is divided into 14 defenses: acting out, splitting, displacement, fantasy, omnipotence, dissociation, projection, repression, undoing, withdrawal, somatization, passive aggression, conversion, sublimation. Factor 2 (F2) represents the global score of mature defense mechanisms, which mitigate unwelcome reality and allow a more adaptive functioning. It consists of seven defenses: altruism, idealization, denial, intellectualization, humor, reaction formation, suppression. The questionnaire has adequate construct validity and internal consistency for all defense mechanisms, whereby all Cronbach’s alpha values are over 0.4 (except passive aggression: α = 0.36). The overall Cronbach’s alpha values for the two factors are 0.84 for F1 and 0.69 for F2 (Steiner et al., 2001). Test–retest reliability ranged from 0.93 for F1 to 0.95 for F2 (Prunas et al., 2019). The Italian version of the questionnaire was used in this study (Prunas et al., 2009). This version has an internal consistency of 0.88 and 0.73 for F1 and F2, respectively (Prunas et al., 2009). Prunas et al. (2014) identified a score of 4.40 as the clinical cut-off only for F1.
(4) The Beck Depression Inventory (BDI-II) (Beck et al., 1996) contains 21 items designed to measure cognitive, affective, and somatic symptoms associated with depression. The BDI-II was designed to correspond closely with Diagnostic and Statistical Manual of Mental Disorders, Fourth Edition, Text Revision (DSM-IV-TR) diagnostic criteria for major depressive disorder. There are four possible choices for each question with answers receiving either 0, 1, 2, or 3 points. Higher scores are indicative of higher self-reported depressive symptomatology. The test–retest reliability is reported to be ≥0.90 (Beck et al., 1996). The BDI-II showed Cronbach’s α of 0.93 for non-clinical samples and test–retest reliability of 0.93 at 1 week (Arbisi and Farmer, 2001). Different severity levels have been defined on an empirical basis (Dozois et al., 1998): minimum depression (scores of 0 to 13); mild depression (scores of 14 to 19); moderate depression (scores of 20 to 28); severe depression (scores of 29 to 63). The Italian version of the questionnaire was used in this study (Ghisi et al., 2006). The Italian version of the questionnaire (Ghisi et al., 2006), which was used in this study, demonstrated a good internal consistency, with Cronbach’s alpha coefficient of 0.80 (Ghisi et al., 2006).
(5) The State-Trait Anxiety Inventory-Y Form (STAI-Y) (Spielberger et al., 1983) measures severity of anxiety symptoms and differentiate acute (state) from chronic (trait) anxiety. The STAI-Y is composed of 40 questions that are answered using a 4-point Likert-type scale. Scores are grouped into three categories (Elliott, 1993): low anxiety (scores of 20 to 39), medium anxiety (scores of 40 to 59), and high anxiety (scores of 60 to 80). The STAI-State test–retest reliability has been reported as 0.40 and the Trait test–retest reliability has been reported as 0.86 (Rule and Traver, 1983). The Cronbach’s α ranged from 0.83 to 0.92 for State scores and 0.86 to 0.92 for Trait scores (Dreger and Katkin, 1985). The Italian version of the questionnaire was used in this study (Pedrabissi and Santinello, 1989). For the Italian version, the internal consistency coefficients for the state anxiety scale range from 0.91 to 0.95 (depending on the sample) and for the trait anxiety scale they range from 0.85 to 0.90 (Pedrabissi and Santinello, 1989).

In order to allow for comparisons with the categories identified in the literature, we grouped subscales scores into three categories: low, medium, and high level. For TCI-R scores, low values correspond to low and significantly low values defined on the raw scores in Martinotti et al. (2008), while high values correspond to high and significantly high values (Martinotti et al., 2008). For the BDI-II, medium values correspond to mild and moderate values defined in Dozois et al. (1998). For REM-71 F1, no medium range is defined in Prunas et al. (2014), therefore we only classified scores into low and high level according to the clinical cut-off. The scores of the STAI-Y are already grouped into low, medium and high anxiety.

### Statistical Analysis

Continuous variables have been reported as mean, standard deviation and quartiles, while categorical variables have been described in terms of frequency distribution.

Cronbach’s α was computed to assess the internal consistency of each psychometric scale. The values of the psychometric scales were compared with normative data published on the Italian population, by means of the non-parametric Wilcoxon’s test. Comparisons of the distribution of the psychometric scales between two groups were performed with Mann-Whitney’s test. In both types of analyses, *p*-values were adjusted with Bonferroni’s correction to account for multiple testing.

*P*-values less than 0.05 were considered significant. All statistical analyses were carried out with the Statistical Package for Social Science version 21.0 (SPSS Inc., Chicago, IL, United States) and R 3.5.0^1^.

## Results

Detailed descriptive statistics are reported in Table 1. The analyzed sample is composed of 52 women (mean age 30.29 ± 5.58 years, range 19–39 years), suffering from various oncological malignancies (i.e., 40.38% have hematological cancer, 32.69% have breast cancer, 13.46% have sarcoma, and the remaining 13.47% have other tumors). More than half of the sample (61.54%) had previously undergone surgery. Most of them are in a relationship (76.92%) and do not have children (86.54%). Levels of education include middle school diploma (5.77%), high school diploma (50%), Bachelor’s/Master’s degree (42.31%), Postgraduate degree (1.92%). Most patients work (82.7%).

**TABLE 1:** Descriptive statistics of the socio-demographic and clinical characteristics of the sample (*n* = 52).

	**Variable**	**Mean (SD)**	**Median [IQR]**
	**Age, years**	**30.29 (5.58)**	**31.00 [26.35–34.75]**
**Variable**		**Frequency**	**Relative Frequency (%)**
Marital status	Single	12	23.08%
	In a relationship	27	51.92%
	Married	13	25.00%
Presence of children	Yes	7	13.46%
	No	45	86.54%
Educational level	Middle school	3	5.77%
	High school	26	50.00%
	Bachelor’s/Master’s degree	22	42.31%
	Postgraduate degree	1	1.92%
Occupation	Employee	28	53.85%
	Freelance	15	28.85%
	Housewife	1	1.92%
	Student	8	15.38%
Diagnosis	Hematological cancer	21	40.38%
	Breast cancer	17	32.69%
	Sarcoma	7	13.46%
	Brain cancer	3	5.77%
	Gynecological cancer	2	3.85%
	Melanoma	1	1.92%
	Head and Neck cancer	1	1.92%
Previous surgery	Yes	32	61.54%
	No	20	38.46%
Previous miscarriages	Yes	4 (all voluntary)	7.69%
	No	48	92.31%

The Cronbach’s α coefficient showed good reliability for all psychometric scales (Table 2). As shown in Table 2, means, standard deviations and quartiles were calculated for each of the TCI-R dimensions, for the two factors of the REM-71, for the BDI-II total score, and for the State and Trait anxiety total scores. These values were compared with reference data of the Italian population. Wilcoxon test indicated significantly lower scores for the TCI-R dimension of Harm Avoidance (median = 87.50, reference mean value = 96.40, *adj. p* = 0.029) and the STAI-Trait total score (median = 37.00, reference mean value = 42.06, *adj. p* < 0.001). Significantly higher levels were reported for the REM-71 mature defense mechanisms (median = 5.86, reference mean value = 5.22, *adj. p* < 0.001). Wilcoxon test also indicated higher scores for the TCI-R dimension of Persistence (median = 123.00, reference mean value = 116.30, *p* = 0.005) and Self-Directedness (median = 146.00, reference mean value = 139.10, *p* = 0.005). However, the corresponding *p*-values adjusted with Bonferroni’s correction resulted to be slightly higher than the defined significance level (*adj. p* = 0.055, *adj. p* = 0.059, respectively for Persistence and Self-Directedness).

**TABLE 2:** Descriptive statistics of the questionnaires and comparison with normative data.

**Variable**	**Cronbach’s α**	**Mean (SD)**	**Median [IQR]**	**Reference mean value**	***p*-value**	**Adj. *p*-value**
NS TOT	0.7888	102.85 (12.64)	101.00 [95.00–111.50]	98.50	0.041	0.489
HA TOT	0.8982	88.46 (17.37)	87.50 [76.00–101.25]	96.40	0.002	**0.029**
RD TOT	0.7700	104.85 (10.56)	103.00 [97.00–112.00]	101.40	0.043	0.512
PS TOT	0.9143	123.00 (16.41)	123.00 [111.75–134.50]	116.30	0.005	0.055
SD TOT	0.8721	145.44 (15.82)	146.00 [138.00–158.25]	139.10	0.005	0.059
CO TOT	0.8231	136.48 (12.05)	135.50 [129.25–146.75]	134.90	0.384	1.000
ST TOT	0.8637	67.23 (14.94)	66.50 [55.00–78.75]	69.90	0.289	1.000
REM-71 F1	0.8876	3.76 (0.93)	3.64 [3.23–4.21]	3.66	0.788	1.000
REM-71 F2	0.7445	5.82 (0.84)	5.86 [5.43–6.46]	5.22	<0.001	**<0.001**
BDI-II	0.8297	9.06 (6.07)	8.50 [5.00–12.00]	7.79	0.151	1.000
STAI-State	0.9405	45.02 (11.30)	42.50 [37.00–54.00]	39.62	0.008	0.093
STAI-Trait	0.8406	36.31 (6.71)	37.00 [31.25–39.00]	42.06	<0.001	**<0.001**

*NS, Novelty Seeking; HA, Harm Avoidance; RD, Reward Dependence; PS, Persistence; SD, Self-Directedness; CO, Cooperativeness; ST, Self-Transcendence; REM-71, Response Evaluation Measure; F1, Factor 1; F2, Factor 2; BDI-II, Beck Depression Inventory-II; STAI, State-Trait Anxiety Inventory. The bold values indicate significant differences after Bonferroni’s correction.*

Mann–Whitney’s test was used to compare the distribution of the psychometric scales between the two groups defined by age, according to literature indicating 35 years as the cut-off for advanced reproductive age (e.g., Klein and Sauer, 2001; Cobo et al., 2018). Only Factor 1 of the REM-71 was significantly different between the two groups, suggesting that younger women use immature defense mechanisms to a greater extent (median [IQR] in age ≤35 years = 3.82 [3.41–4.30] vs. 3.18 [2.38–3.54] in age >35 years, *p* = 0.003, *adj. p* = 0.036).

Finally, the scores reported by the patients in these scales have been classified in low, medium and high according to cut-offs reported in the validation studies (see Table 3). Most patients report low scores on the REM-71 Factor 1 (82.7%), BDI-II total score (86.5%), and STAI-Trait scale (76.9%). Predominantly medium scores have been obtained on the Novelty Seeking total score (82.7%), Harm Avoidance total score (65.4%), Reward Dependence total score (86.5%), Persistence total score (71.2%), Self-Directedness total score (78.9%), Cooperativeness total score (88.5%), Self-Transcendence total score (65.4%), and STAI-State scale (51.9%).

**TABLE 3:** Classification of the scores according to the cut-offs identified in the literature.

**Variable**	**Low scores**	**Medium scores**	**High scores**
NS TOT	2 (3.8%)	43 (82.7%)	7 (13.5%)
HA TOT	12 (23.1%)	34 (65.4%)	6 (11.5%)
RD TOT	1 (2.0%)	45 (86.5%)	6 (11.5%)
PS TOT	2 (3.8%)	37 (71.2%)	13 (25.0%)
SD TOT	2 (3.8%)	41 (78.9%)	9 (17.3%)
CO TOT	2 (3.8%)	46 (88.5%)	4 (7.7%)
ST TOT	8 (15.4%)	34 (65.4%)	10 (19.2%)
REM-71 F1^†^	43 (82.7%)	–	9 (17.3%)
BDI-II	45 (86.5%)	7 (13.5%)	–
STAI-State	18 (34.6%)	27 (51.9%)	7 (13.5%)
STAI-Trait	40 (76.9%)	12 (23.1%)	–

*^†^A clinical cut-off for the REM-71 was available only for F1 (see Prunas et al., 2014). NS, Novelty Seeking; HA, Harm Avoidance; RD, Reward Dependence; PS, Persistence; SD, Self-Directedness; CO, Cooperativeness; ST, Self-Transcendence; REM-71, Response Evaluation Measure; F1, Factor 1; BDI-II, Beck Depression Inventory-II; STAI, State-Trait Anxiety Inventory.*

## Discussion

As far as we know, no studies have investigated personality characteristics and defensive style of women who are motivated to undergo fertility preservation following cancer diagnosis. The purpose of this study was therefore to analyze the personality profile of female patients referred to the Oncofertility Unit after cancer diagnosis and prior to gonadotoxic treatment.

As hypothesized, our findings suggest that patients who are willing to undergo fertility preservation display characteristics that may favor psychological adjustment to cancer.

Concerning personality features, the lower scores of Harm Avoidance obtained by our sample of patients compared to normative data (Martinotti et al., 2008) may favor a better adjustment to the disease, promoted by optimism, courage and energy in facing new challenges. Moreover, our findings show that our patients tend to display higher levels of Persistence and Self-Directedness. Although these results need to be confirmed in a bigger sample, these scores may imply the tendency to maintain a behavior in spite of intermittent reinforcement, being perseverant in front of frustration and fatigue (Persistence); and personal integrity and efficacy, responsibility, goals for the future, constructiveness and hope (Self-Directedness). This is important in light of the results of other studies showing that low levels of Harm Avoidance (Bonacchi et al., 2012) and high levels of Self-Directedness (Bonacchi et al., 2012; Honorato et al., 2017) are significantly associated with a better quality of life in cancer patients. This may be associated with a greater ability to adjust to the disease. Contrarily to our expectations, our patients did not significantly differ from normative data (Martinotti et al., 2008) in their levels of Reward Dependence, as most of them exhibited medium scores in this subscale.

Moreover, the present findings show that our participants tend to use mature defense mechanisms to a greater extent than the general population (Prunas et al., 2009). In front of a stressful and destabilizing condition such as cancer diagnosis, patients who are willing to undergo fertility preservation may mobilize skills that allow to contain the negative effects of such experience and to manage it in the most functional way, at least in the initial stage of their treatment. Other research has shown that primitive defense mechanisms, such as repression, displacement, projection and regression, predict worse psychological adjustment in oncological patients, in terms of greater distress 1 year after diagnosis (Hyphantis et al., 2011) and long-term vulnerability to the development of anxiety (Månsson et al., 1998). Emotional suppression, considered as an immature defense mechanism, has been found to predict chemotherapy symptomatic side effects and unpleasant mood states in samples of breast cancer patients (Iwamitsu et al., 2005; Schlatter and Cameron, 2010). Accordingly, a review focusing on oncological patients points out that mature defenses are associated with higher physical and emotional functioning, whereas mental inhibition defenses, in particular repression, foster psychosomatic symptoms, passive decisional preferences and worse physical and emotional health (Di Giuseppe et al., 2018).

Finally, none of our patients displayed severe symptoms of depression or elevated levels of trait anxiety. Notably, a meta-analyisis showed that among oncological patients pooled prevalence of depression and anxiety disorders is, respectively, 16.5 and 9.8% (Mitchell et al., 2011). In addition, lifetime prevalence of depression and anxiety disorders is also higher in Italian community samples, corresponding, respectively, to 10.1 and 11.1% (De Girolamo et al., 2006). Indeed, the decision to compare this sample of patients with normative data derived from the general population, rather than referring to other oncological patients, is attributable to the fact that these patients are mainly in the initial stage of their treatment and cancer has still not imposed many limitations to their daily life (which instead may contribute to a higher prevalence of depression and anxiety observed during treatments). These findings concerning psychopathological symptoms further support the high functioning profile of our patients. However, a few patients (13.5% of the sample) exhibited high scores of state anxiety, probably as an acute reaction to a threatening event.

Cancer exposes young women to a life crisis in two respects: the diagnosis itself and the threat of impaired fertility due to treatment (Tschudin and Bitzer, 2009). In fact, fertility concerns may disrupt future family planning potential, leading to psychological distress (Crawshaw, 2013). However, patients often feel uncomfortable expressing their fertility concerns as they are confronted with an uncertain future (Hershberger et al., 2013a). Moreover, cancer diagnosis requires choosing among intensive treatment options, leaving patients frequently submerged by the complex medical information they have to process in a very short time (Hershberger et al., 2013b). In addition, in order to prevent delays in the beginning of therapy, fertility preservation is proposed to patients shortly after they have received the diagnosis of cancer. Comprehensibly, these women can be emotionally overwhelmed, thus the ability to make long-term plans should not be taken for granted. Scheduling future childbearing may subtend the faculty to prefigure survival and picture oneself as a parent, which can be promoted by adaptive personality traits. Moreover, awareness about the uncertainty of the future may also be considered as an effective competence for future mothers, since they may face more realistically such a big project in their life and in their family life.

Some limitations of the present research must be acknowledged. First, a larger, more representative sample would increase the generalizability of the results. However, not all oncological patients of childbearing age can undergo fertility preservation due to several factors, including limited time available to make decisions about their reproductive health before the start of antineoplastic treatments, and lack of referral from their oncologists. Second, the cross-sectional design of the study did not allow testing the stability of the results nor the determination of the causal relations among variables. Nevertheless, longitudinal data about TCI-R (Martinotti et al., 2008) and REM-71 (Prunas et al., 2019) support a good stability of the scores over time. Third, the lack of a control group prevents drawing conclusions concerning the features of patients who refuse to inquire about fertility preservation. Despite the comparison with normative data clearly shows the adaptive profiles of our sample, we are not able to completely rule out that these features characterize young women facing cancer diagnosis. However, previous works show significant variation in psychological reaction and adjustment to chronic illnesses (Stanton et al., 2007) and, in particular, to cancer (Infurna et al., 2013).

In spite of these limitations, our study is relevant for several reasons. First, participants were recruited face-to-face in a clinical setting, allowing us to sample all the patients that showed up to the Oncofertility Unit. Second, we assessed patients’ will to undergo specific preservation techniques without focusing on the outcomes of the procedure itself, which, in some cases, do not correspond to their decision (Melo et al., 2019). In addition, most previous studies used retrospective designs, whereas we recruited patients before the fertility preservation.

### Clinical Implications and Conclusion

The current study is the first to investigate the personality profile of oncological patients who are willing to undergo fertility preservation. Our findings suggest that these women display functional personality traits and defense style, in association with low levels of depression and trait anxiety. These features may enable a proactive attitude to cancer and the ability to make long-term plans.

However, it is possible that oncologisists refer to Oncofertility Units only those patients who do not seem too emotionally overwhelmed and thus appear able to bear what fertility preservation procedures entail. An empirical understanding of these features could allow identifying women who may be more at risk of facing higher difficulties in the process of adjustment to their disease. This could help clinicians in choosing to dedicate more time to certain patients to explain the advantages of fertility preservation, fostering targeted interventions.

## Data Availability Statement

The datasets presented in this article are not readily available because participants did not provide written informed consent for it. Requests concerning the datasets should be directed to g.perego23@campus.unimib.

## Ethics Statement

The studies involving human participants were reviewed and approved by San Raffaele Hospital Ethics Committee (protocol N. 149/INT/2019). The patients/participants provided their written informed consent to participate in this study.

## Author Contributions

VEDM, LC, GP, PT, and GM contributed to conception and design of the study. PT and PMVR organized the database and wrote sections of the manuscript. PMVR performed the statistical analysis. GP wrote the first draft of the manuscript. VEDM, GM, VS, AB, and MC commented on previous versions of the manuscript. All authors contributed to manuscript revision, read, and approved the submitted version.

## Conflict of Interest

The authors declare that the research was conducted in the absence of any commercial or financial relationships that could be construed as a potential conflict of interest.

## Footnotes

1 http://www.R-project.org/
